# Children in reviews: Methodological issues in child-relevant evidence syntheses

**DOI:** 10.1186/1471-2431-5-38

**Published:** 2005-09-21

**Authors:** Kristie Cramer, Natasha Wiebe, Virginia Moyer, Lisa Hartling, Katrina Williams, George Swingler, Terry P Klassen

**Affiliations:** 1 Alberta Research Centre for Child Health Evidence, Department of Pediatrics, University of Alberta, Edmonton, Alberta, Canada; 2 Department of Medicine, Division of Nephrology, University of Alberta, Edmonton, Alberta, Canada; 3 Center for Population and Evidence Based Medicine, University of Texas-Houston Health Sciences Center, Houston, Texas, USA; 4 Department of Pediatrics and Child Health, The Children's Hospital at Westmead, Westmead, New South Wales, Australia; 5 School of Child and Adolescent Health, University of Cape Town, Cape Town, South Africa

## Abstract

**Background:**

The delivery of optimal medical care to children is dependent on the availability of child relevant research. Our objectives were to: i) systematically review and describe how children are handled in reviews of drug interventions published in the Cochrane Database of Systematic Reviews (CDSR); and ii) determine when effect sizes for the same drug interventions differ between children and adults.

**Methods:**

We systematically identified all of the reviews relevant to child health in the CDSR 2002, Issue 4. Reviews were included if they investigated the efficacy or effectiveness of a drug intervention for a condition that occurs in both children and adults. Information was extracted on review characteristics including study methods, results, and conclusions.

**Results:**

From 1496 systematic reviews, 408 (27%) were identified as relevant to both adult and child health; 52% (213) of these included data from children. No significant differences were found in effect sizes between adults and children for any of the drug interventions or conditions investigated. However, all of the comparisons lacked the power to detect a clinically significant difference and wide confidence intervals suggest important differences cannot be excluded. A large amount of data was unavailable due to inadequate reporting at the trial and systematic review level.

**Conclusion:**

Overall, the findings of this study indicate there is a paucity of child-relevant and specific evidence generated from evidence syntheses of drug interventions. The results indicate a need for a higher standard of reporting for participant populations in studies of drug interventions.

## Background

Health care decisions for individual patients are influenced by the availability of evidence that pertains most directly to the patient. However, health care providers are often faced with a paucity of evidence for specific patient groups such as children and youth, and thus must rely on evidence of questionable applicability as the basis for their health care decisions.

In the case of children, where there is a recognized gap in research evidence [1-4], health care providers frequently extrapolate evidence derived from adult studies to guide their decision-making [5]. To illustrate, several European surveys found 29–72% of the drugs prescribed to children are unlicensed or off-label [6-8]. In the United States 80% of the new drugs approved between 1984 and 1989 had no indication for use in children [9]. As a result, children may be less likely than adults to receive health care that is based on research in children.

This is unsettling given that it is widely recognized children and adults differ [10] in terms of their physiology and biology as well as the developmental and disease processes they experience [11,12]. Children and adults may also differ with respect to their response to therapies. For example, selective serotonin reuptake inhibitors have been found to be effective for treating depression in adults, whereas the evidence suggests these drugs are associated with an increased risk of suicidal behaviour in children [13]. Children treated with glucocorticosteroids for long periods of time have been found to be at risk of growth retardation, whereas this risk does not exist in adults [14]. Further, phenobarbital has a sedative effect in adults but may cause paradoxical hyperactivity in children [15]. These examples illustrate the potential risks of treating children without child-specific evidence. Nevertheless until pediatric research is conducted, health care providers are faced with a serious dilemma. If they deny children treatments known to be effective in adults, they may deny them effective treatments. Alternatively, if they treat children and/or youth with untested interventions they may be using treatments that differ in effect or are even harmful.

Health care providers increasingly turn to evidence syntheses to guide their clinical decisions. Systematic reviews represent the most rigorous and comprehensive synthesis of information on a specific clinical question. The Cochrane Collaboration is an international organization that promotes rigorous methodological standards in the preparation and maintenance of systematic reviews and supports the use of evidence by ensuring systematic reviews are available through the Cochrane Database of Systematic Reviews (CDSR). The CDSR presents a sample of high quality systematic reviews upon which to base methodological investigations [16]. We sought to systematically identify all child-relevant reviews published in the CDSR and describe whether child specific evidence was available. We then examined whether adults and children differed with respect to their response to different therapies.

## Methods

### Systematic review identification

One investigator reviewed the titles and abstracts of all 1496 complete systematic reviews published in the CDSR 2002, Issue 4 and identified relevant reviews in which: the efficacy or effectiveness of a drug intervention was investigated and the condition investigated occurs in both children and adults. Reviews of conditions judged by a pediatrician on our research team (TK) to be rare in children were excluded. If there was any uncertainty about the relevance of a review a pediatrician from our research team was consulted.

### Data extraction

Characteristics of each systematic review were extracted using a standard data collection form. Information was extracted on review characteristics such as study inclusion criteria, number of included trials, results, and author's conclusions (complete list in Appendix A).

### Descriptive analysis

Each systematic review was categorized as "adult", "child", or "mixed" based on the trials the review authors ***intended ***to include and the trials that were ***actually ***included. If the review authors did not specify the population, the ***intended ***review type was categorized as 'not specified'. If the ages of the trial participants could not be determined, the ***actual ***review type was categorized as 'uncertain'.

### Quantitative data analysis: Effect size differences between children and adults

Numerical results were analyzed in Stata 7.0 and S-plus 6.0. The primary outcome for our analysis was the primary outcome investigated in the review. If the systematic review authors did not specify a primary outcome, the first outcome listed by the review authors was used. Effect sizes (e.g., relative risks and standardized mean differences) and their corresponding standard errors were calculated for each trial included in each review using the data as presented in the review. Adults were defined as 18 years and older and children were defined as 18 years and younger.

Results were combined separately for each review using a random effects model [17]. For dichotomous results, summary relative risks (RR) were calculated separately for children and adults. Then, ratios of RRs (i.e., child RR divided by adult RR) were estimated using meta-regression [18] to summarize the relationship between child and adult RRs (i.e., to investigate potential effect size differences between adults and children). The natural logarithm of the RR responses is regressed on a child indicator variable; the exponentiated estimated coefficient is the ratio of RR. For drug interventions intended to prevent an adverse outcome, a ratio of RRs less than 1 indicates that children experienced more benefit from the drug intervention than adults. For drug interventions intended to bring about a beneficial outcome, a ratio of RRs less than 1 indicates children experienced less benefit from the drug intervention than adults. A ratio of RR of 0.75 or 1.33 was considered a "small" but clinically relevant difference and 0.5 or 2 was considered to be a "moderate" difference. Ninety-five percent confidence intervals (95% CI) were calculated for each summary statistic.

For reviews reporting continuous variables, standardized mean differences (SMD) were calculated separately for adults and children. Differences of SMDs (child SMD minus adult SMD) were calculated using meta-regression to summarize the relationship between child and adult SMDs (i.e., to investigate potential effect size differences between adults and children). The SMD responses are regressed on a child indicator variable; the estimated coefficient is the difference in SMD. A difference in SMDs of ± 0.2 was considered a "small" but clinically relevant difference [19] and of ± 0.5 was considered to be a "moderate" difference. Ninety-five percent confidence intervals (95% CI) were calculated for each summary statistic.

Heterogeneity was quantified for the overall summary estimate using the I^2 ^statistic [20,21], that indicates the percent variability due to between-study variability as opposed to within-study variability. An I^2 ^value greater than 50% was considered moderately large.

## Results

### Description of child relevant reviews

Of the 1496 completed reviews in the CDSR, 403 were considered relevant to child health. Five of the relevant reviews investigated both treatment and preventive interventions for the same condition; each of these was counted as two independent reviews. Therefore, 408 reviews published by 34 different Cochrane Collaborative Review Groups (list available on request) were identified and evaluated.

### Intended review types

In 16% (67/408) of reviews, the authors intended to only include adult trials; 11% (45/408) intended to only include child trials; 38% (153/408) intended to include both; and 35% (143/408) did not specify age criteria.

Thirty-six percent (24/67) of the adult review authors defined individuals 14 and older as adults, 43% (29/67) did not provide a definition for "adult", and the remaining 21% defined adults as 18 and older. Four percent (2/45) of the child review authors defined children as 19 years old and younger, 9% (4/45) did not provide a definition for "children", and the remaining 87 % (39/45) defined children as 18 years old and younger. The majority (89%) of the mixed review authors stated they planned to include children and adults but they did not define "children" or "adults". The remaining 11% (16/153) stated they would include individuals greater than one month.

### Actual review types

Fifty-five percent (37/67) of the ***intended ***adult reviews ***actually ***included adult trials; 80% (36/45) of the ***intended ***child reviews ***actually ***only included child trials; and 63% (96/153) of the ***intended ***mixed reviews ***actually ***included both adult and child trials and/or mixed trials. The types of trial participants were could not be determined in 35% (50/143) of the not specified reviews (Table 1).

**Table 1:** Intended review type versus actual types of included trials

		**Intended Review Type**				
		**Adult Reviews ** **N = 67 (%)**	**Child Reviews ** **N = 45 (%)**	**Mixed Reviews ** **N = 153 (%)**	**Not specified Reviews ** **N = 143 (%)**	**Total ** **N = 408 (%)**

**Actual type of included trials**	**Adult only**	37 (55)	0 (0)	17 (11)	24 (17)	78 (19)
	**Child only**	0 (0)	36 (80)	8 (5)	6 (4)	50 (12)
	**Mixed**	9 (13)	7 (16)	96 (63)	51 (36)	163 (40)
	**Uncertain**	18 (27)	2 (4)	24 (17)	50 (33)	94 (23)
	**No trials**	3 (4)	0 (0)	8 (5)	12 (8)	23 (6)

### Characteristics of the reviews

Table 2 outlines the characteristics of the reviews by ***intended ***review type. In 6% (23/408) of the reviews, no relevant studies were found. The remaining 94% (385/408) included a median of 8 (interquartile range (IQR) 4–15) trials. The number of participants included in each review could be determined in 91% (352/385) of the reviews; they included a median of 681 (IQR 210–1670) participants.

**Table 2:** Characteristics of the systematic reviews by *intended *review type

	***Intended *Review Type**				
**Variable**	**Adult Reviews ** **N = 67**	**Child Reviews ** **N = 45**	**Mixed Reviews** ** N = 153**	**Not Specified Reviews ** **N = 143**	**Total ** **N = 408**

Funding, N (%)					
External	26 (39)	23 (51)	67 (44)	89 (62)	205 (50)
Internal	56 (84)	24 (53)	92 (60)	93 (65)	265 (65)
No funding	6 (9)	10 (22)	45 (29)	22 (15)	83 (20)
					
Languages of studies, N (%)					
English only	1 (1)	0	0	3 (2)	4 (1)
English and non-English (no language restrictions)	34 (51)	16 (36)	53 (35)	54 (38)	157 (38)
Not specified	32 (48)	29 (64)	100 (65)	86 (22)	247 (61)
					
Included study designs, N (%)					
RCT	47 (70)	30 (67)	91 (60)	82 (57)	250 (61)
CCT	20 (30)	15 (33)	62 (40)	60 (42)	157 (38)
Other	0	0	0	1 (0.7)	1 (0.2)
					
Median number of included trials, Median (Interquartile range)	9 (3–14)	10 (5–13)	8 (3–12)	7 (4–17)	8 (4–15)
					
Median number of participants, Median (Interquartile range)	813 (263–1836)	718 (313–1712)	551 (235–1474)	776 (305–2151)	681 (210–1670)
					
Number of reviews where the authors planned a subgroup analysis by age, N	2	10	30	10	52
					
Number of reviews where authors conducted a subgroup analysis by age, N	2	6	20	3	31
					
Number of reviews where authors found a difference in effect between adults and children, N	1	0	0	0	1

Thirteen percent (52/408) of the review authors planned to conduct a subgroup analysis based on age. Sixty percent (31/52) of these conducted the planned subgroup analysis and 3% (1/31) of these found a significant effect size difference by age. Only 11 of these 52 reviews specified the age groups they planned to compare: older adults to younger adults (1), children under 5 to adults and children over 5 (1), children and adults to older adults (1), children less than 12 to adolescents and adults older than 12 (1), children (0–12 years old) to adolescents (12–18) (2), and adults (18+) to children (<18)(5). None of these comparisons were significant. One unplanned subgroup analysis by age found a significant difference but the age used for the subgroup analysis was not reported.

### Quantitative data analysis: Effect size differences between children and adults

**Figure 5:**
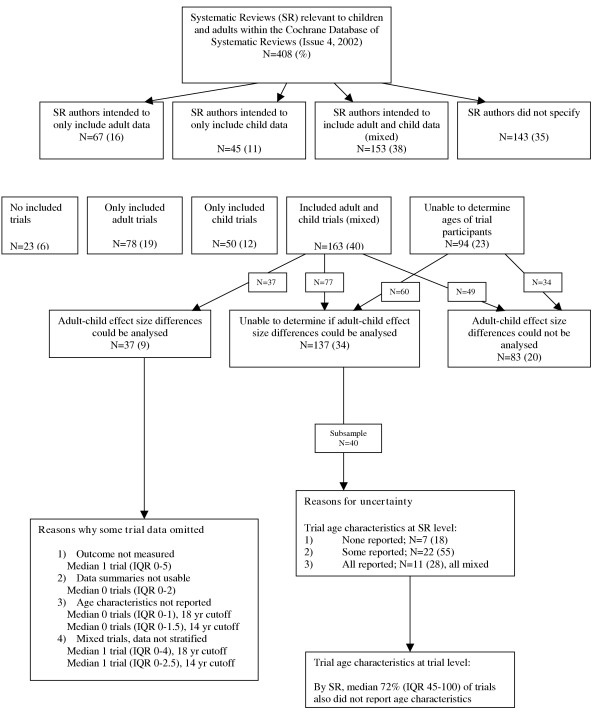
Flow of data loss.

Only 9% (37/408) of the reviews included enough data to allow for an investigation of effect size differences between adults and children. Figure 5 depicts the reasons that data from the majority of the reviews could not be used for this investigation. Thirty-seven percent of the reviews had no comparative data; the remaining 54% of reviews could not be used because of insufficiencies in the reporting of participant characteristics from the included trials.

None of these reviews reported separate child and adult data collected from the same trial, which is the most likely to provide valid information as both adults and children would have been exposed to the same experimental procedures [22]. As a result, our analyses were exclusively based on between study comparisons where the adults and children may have been exposed to different experimental procedures [22]. Among the 37 reviews, a median of two studies (IQR 1 to 5) per review were omitted from the meta-regression because these studies either only presented collapsed adult and child data or they did not define the age ranges of the participants (Figure 5).

**Figure 1:**
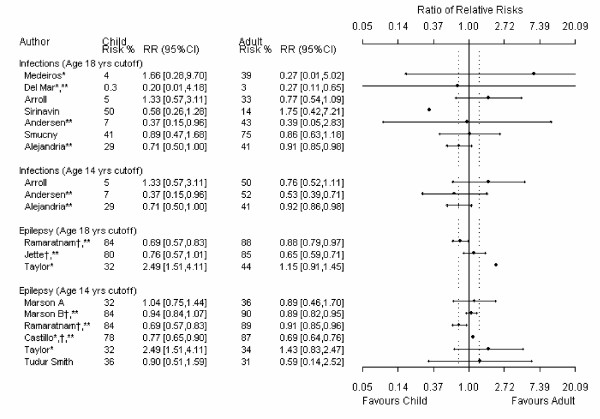
**Meta-metagraph comparing child and adult relative risk summaries**. * I^2 ^for the overall relative risk is greater than 50%. ** the overall relative risk was statistically significant. ^† ^an event in the original data indicated benefit; the data has been manipulated so that an event indicates harm.

**Figure 2:**
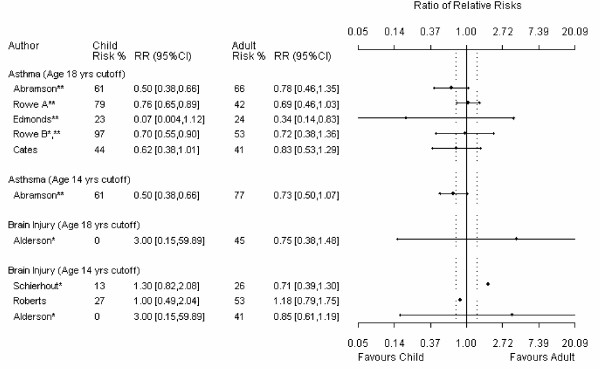
**Meta-metagraph comparing child and adult relative risk summaries, continued**. * I^2 ^for the overall relative risk is greater than 50%. ** the overall relative risk was statistically significant. ^† ^an event in the original data indicated benefit; the data has been manipulated so that an event indicates harm.

**Figure 3:**
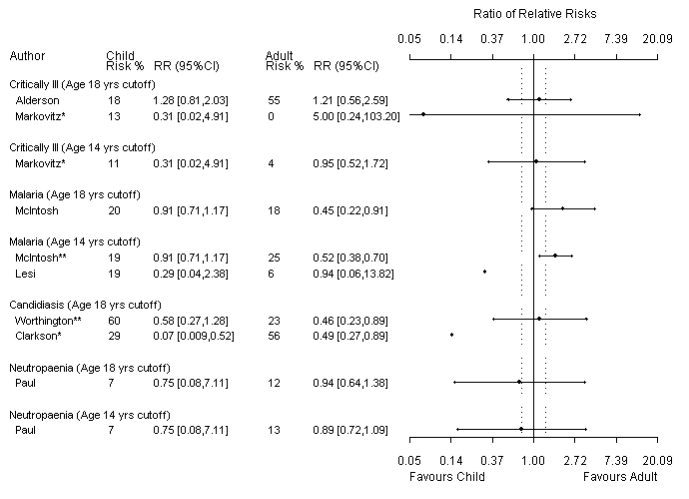
**Meta-metagraph comparing child and adult relative risk summaries, continued**. * I^2 ^for the overall relative risk is greater than 50%. ** the overall relative risk was statistically significant. ^† ^an event in the original data indicated benefit; the data has been manipulated so that an event indicates harm.

**Figure 4:**
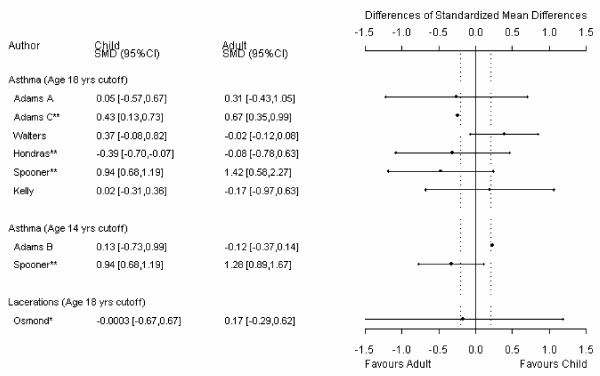
**Meta-metagraph comparing child and adult SMD summaries**. * I^2 ^for the overall relative risk is greater than 50%. ** the overall relative risk was statistically significant. ^† ^a greater value in the original data indicated less benefit; the data has been manipulated so that a greater value indicates more benefit.

For the main comparison in each review, 24% (9/37) had useable data for only one child and one adult trial. Because at least 3 data points are required, data from these meta-analyses could not be analyzed using meta-regression and precision around these estimates is absent from the meta-metagraphs (Figures 1, 2, 3, 4).

The age definitions of adults and children were not consistent across reviews. A sensitively analysis was therefore conducted in which children were defined as 14 years and younger and adults were defined as 14 years and older. There were mostly negligible differences between the different age cutoff estimates.

When we compared the magnitude of effect between adults and children, only one (1/37) estimate was significant; this favored adults [23]. This estimate was significant for the 14-year cut off (14 studies, 947 patients) but not for the 18-year cutoff (8 studies, 1405 patients). In this review any artemisinin drug was compared to standard treatment (*e.g.*, quinoline drugs) for severe malaria as measured by mortality.

Four of the 37 meta-analyses measured therapeutic benefit instead of prevention. To create a consistent definition for direction in the meta-metagraphs, RR for prevention instead of therapy was measured for these four meta-analyses. When the inverse was used one of these meta-analyses [24] showed a significant difference between adults and children for both age cutoffs; the results favored children [25]. This meta-analysis compared add-on lamotrigine to placebo in drug resistant partial epilepsy as measured by treatment success; we measured treatment failure. However, if the outcome in this meta-analysis is left as a therapeutic outcome (as presented in the review) the ratio of RRs for both age cutoffs is not significant. Four studies from this meta-analysis (538 patients) were included in the 18-year subgroup comparison and ten studies (797 patients) were included in the 14-year subgroup comparison. Ten studies (425 patients) were omitted from the 18-year subgroup comparison and nine studies (403 patients) were omitted from the 14-year subgroup comparison due to undefined age ranges.

Aside from the two meta-analyses discussed above, a significant difference between adult and child responses to therapy was not found in the majority (35/37) of the meta-analyses. However, in all but one of the comparisons [26] contained "small" effect sizes within the plausible values of the 95% confidence limits. Of the comparisons that used the 18-year cut off, 59% (17/29) had point estimates that were larger than the "small" effect size cutoff and 28% (8/29) had point estimates that were larger than the "moderate" effect size cutoff. Of the comparisons that used the 14-year cut off, 63% (12/19) had point estimates that were larger than the "small" effect size cutoff and 11% (2/19) had point estimates that were larger than the "moderate" effect size cutoff. In addition, of the meta analyses that included enough data points to be analyzed using meta-regression (N = 25), 84% (21/25) at the 18 year cut off and 79% (11/14) at the 14 year cut off had 95% confidence intervals that contained "moderate" effect sizes.

## Discussion

This study provides support for the speculation there is too little child health evidence available from systematic reviews published in the CDSR. Although all 408 of the reviews evaluated here were on topics relevant to children, only about half ***intended ***(48%) or ***actually ***(52%) included data from children. Even when reviews included evidence generated from children, they frequently did not distinguish between children and adults in the analysis. Of the reviews that planned to include both children and adults (39%) only 20% planned a subgroup analysis by age. When data were available to investigate effect size differences between adults and children, the vast majority of comparisons lacked the power to detect "small" but clinically important differences.

Of particular interest was the finding that substantial variability occurred among the reviews with respect to how children, adolescents, and adults were defined. Some of the reviews defined adults as 14 and older whereas others defined them as 19 and older. The participants were often only described as "adults", "children", or "school-aged children". Furthermore, several reviews did not provide any information about the ages of the participants in the included studies. For example, 18% of the mixed reviews did not report the ages of the participants in any of the included studies and 55% only reported the ages of the participants in some of the included studies. Perhaps this is because ages were not reported at the trial level. We reviewed the included studies in a random sub-sample of the reviews (N = 40) and found when age characteristics were not reported in the review, a median of 72% of the trial authors also did not report age characteristics in the trial (Figure 5). Poor reporting decreases the amount of data available for a quantitative subgroup analysis in evidence reviews and limits the generalizability of results by health care professionals. The CONSORT statement for reporting of RCTs delineates appropriate reporting of baseline demographics [27]. Similarly, for meta-analyses QUOROM, MOOSE and the Cochrane handbook for systematic reviews of interventions provide guidelines for appropriate reporting of study characteristics including a description of the participant populations [28-30].

Our comparisons lacked the power to detect "small" and most "moderate" clinically significant differences. Several factors contribute to this lack of power: 1) too few studies; 2) trial authors did not report age characteristics of the participants, decreasing the number of useable studies; 3) systematic review authors did not report age data decreasing the number of useable studies; and 4) segregated adult and child data was not presented in the mixed trials. Until there is clear evidence whether there are or are not differences between adults and children, separate analyses need to be conducted, particularly where there is biological plausibility for a difference.

Our study was limited by the available data. Because the definitions for adult and child varied among the reviews, standard definitions for child (0–18) and adult (18+) could not be used consistently to categorize the reviews. Instead, the categories were based on the review author's definitions, resulting in less precise categories. In addition, since we did not review psychosocial and educational interventions the results of this study cannot be applied to these interventions. We also did not include reviews of rare conditions because including reviews of these conditions (frequently recognized as different in children and adults) had the potential to bias the results in favour of our hypothesis. Finally, because we only reviewed and summarized systematic reviews published in the CDSR, inferences generated from this study can only be applied to how children are treated in reviews published in the CDSR.

Since within-study evidence was not available for our comparisons, these comparisons are limited by potential between-study confounders [22]. Factors other than the one of interest may be responsible for any of the observed effect size differences, hence these inferences are to be viewed as preliminary, requiring confirmation [22] by trial level child-adult comparisons (*i.e.*, within study comparisons). A potential solution is found in systematic reviews that use individual patient data (IPD). IPD adds more power by controlling for between study confounders and by enabling more sensitive modelling of the age-effect relationship [31]. Standard definitions of "child", "adult", and "adolescent" need to be developed and used in research studies, although caution is indicated to avoid misclassification bias from inappropriate age groupings. Very real biological and physiological differences may be masked by lumping individuals (e.g., adolescents and adults) into the same category.

In addition to including a checklist item for baseline demographics or study characteristics, guidelines developed to improve the quality of reporting studies such as CONSORT, QUOROM, MOOSE, and the Cochrane handbook for systematic reviews of interventions need to specifically suggest standards for reporting participant ages [27-30]. As well, to ensure adequate reporting, investigators need to be aware of and adhere to these accepted checklists and guidelines. Researchers planning to conduct studies that include both adults and children need to consider and investigate the differences between adults and children. We recommend that an *a priori *subgroup analysis by age be included, particularly when there is biological plausibility for differences.

## Conclusion

Our analysis of systematic reviews did not exclude important differences in effect sizes between adults and children. Our research supports the need for better reporting in studies of drug interventions. When combining evidence, researchers need to be aware of the potential differences between the groups they are combining, especially when known physiological and biological differences exist. There is a need to define children, adults, and adolescents and to determine when it is appropriate and necessary to do subgroup analysis by age.

## Competing interests

The author(s) declare they have no competing interests.

## Authors' contributions

All authors contributed towards the conception and design of the study and the interpretation of the data. They also read, edited and approved the final manuscript. KC and TK identified relevant studies. KC, LH and KW participated in the data extraction. NW conceived of and conducted the statistical analysis. KC conducted the descriptive analysis. KC and NW drafted the manuscript.

## Pre-publication history

The pre-publication history for this paper can be accessed here:



## Supplementary Material

Additional File 1 Appendix A. This is appendix A for the manuscript that provides details of the data extracted from each systematic review included in the study. Click here for file

## References

[B1] Myers RP, Poynard T (2002). Interferon for interferon nonresponding and relapsing patients with chronic hepatitis C. Cochrane Database of Systematic Reviews.

[B2] Panpanich R, Garner P (2002). Antibiotics for treating scrub typhus. Cochrane Database of Systematic Reviews.

[B3] Soares-Weiser K, Brezis M, Leibovici L (2002). Antibiotics for spontaneous bacterial peritonitis in cirrhotics. Cochrane Database of Systematic Reviews.

[B4] Olliaro P, Mussano P (2002). Amodiaquine for treating malaria. Cochrane Database of Systematic Reviews.

[B5] Gilbert RE, See SE, Jones LV, Stanford MS (2002). Antibiotics versus control for toxoplasma retinochoroiditis. Cochrane Database of Systematic Reviews.

[B6] Sipe J, Dunn L (2002). Aciclovir for Bell's palsy (idiopathic facial paralysis). Cochrane Database of Systematic Reviews.

[B7] Lafuente-Lafuente C, Melero-Bascones M (2002). Active chest compression-decompression for cardiopulmonary resuscitation. Cochrane Database of Systematic Reviews.

[B8] Casimiro L, Brosseau L, Milne S, Robinson V, Wells G, Tugwell P (2002). Acupuncture and electroacupuncture for the treatment of RA. Cochrane Database of Systematic Reviews.

[B9] Wen ZH, Gardener E, Wang YP (2002). Nitrates for Achalasia. Cochrane Database of Systematic Reviews.

[B10] Johansen HK, Gotzsche PC (2002). Amphotericin B lipid soluble formulations versus amphotericin B in cancer patients with neutropenia. Cochrane Database of Systematic Reviews.

[B11] Roos YBWEM, Rinkel GJE, Vermeulen M, Algra A, van Gijn J (2002). Antifibrinolytic therapy for aneurysmal subarachnoid haemorrhage. Cochrane Database of Systematic Reviews.

[B12] Elphick H, Southern K (2002). Antifungal therapies for allergic bronchopulmonary aspergillosis in people with cystic fibrosis. Cochrane Database of Systematic Reviews.

[B13] Mwandumba HC, Squire SB (2002). Fully intermittent dosing with drugs for treating tuberculosis in adults. Cochrane Database of Systematic Reviews.

[B14] Meremikwu M, Logan K, Garner P (2002). Antipyretic measures for treating fever in malaria. Cochrane Database of Systematic Reviews.

[B15] Hirst A, Sloan R (2002). Benzodiazepines and related drugs for insomnia in palliative care. Cochrane Database of Systematic Reviews.

[B16] Busch A, Schachter CL, Peloso PM, Bombardier C (2002). Exercise for treating fibromyalgia syndrome. Cochrane Database of Systematic Reviews.

[B17] Lee A, Cooper MC, Craig JC, Knight JF, Keneally JP (2002). Effects of nonsteroidal anti-inflammatory drugs on post-operative renal function in normal adults. Cochrane Database of Systematic Reviews.

[B18] Normand SLT Tutorial in biostatistics meta-analysis: formulating, evaluating, combining, and reporting. Stat Med.

[B19] Cohen J (1988). Statistical Power Analysis for the Behavioural Sciences.

[B20] Higgins JPT, Thompson (2002). Quantifying heterogeneity in a meta-analysis. Stat Med.

[B21] Higgins JPT, Thompson SG, Deeks JJ, Altman DG (2003). Measuring inconsistency in meta-analyses. BMJ.

[B22] Oxman AD, Guyatt GH (1992). A consumer's guide to subgroup analyses. Ann Intern Med.

[B23] McIntosh HM, Olliaro P (2002). Artemisinin derivatives for treating severe malaria. Cochrane Database of Systematic Reviews.

[B24] Ramaratnam S, Marson AG, Baker GA (2002). Lamotrigine add-on for drug-resistant partial epilepsy. Cochrane Database of Systematic Reviews.

[B25] Deeks JJ (2002). Issues in the selection of a summary statistic for meta-anlysis of clinical trials with binary outcomes. Stat Med.

[B26] Marson AG, Kadir ZA, Hutton JL, Chadwick DW (2002). Gabapentin add-on for drug-resistant partial epilepsy. Cochrane Database of Systematic Reviews.

[B27] Altman DG, Schulz KF, Moher D, Egger M, Davidoff F, Elbourne D, Gotzsche PC, Lang T (2001). The Revised CONSORT Statement for Reporting Randomized Trials: Explanation and Elaboration. Ann Intern Med.

[B28] Stroup DF, Berlin JA, Morton SC, Olkin I, Williamson GD, Rennie D, Moher D, Becker BJ, Sipe TA, Thacker SB (2000). Meta-analysis of Obervational Studies in Epidemiology. JAMA.

[B29] Moher D, Cook DJ, Eastwood S, Olkin I, Rennie D, Stroup DF (1999). Improving the quality of reports of meta-analysis of randomized controlled trials: the QUOROM statement. Lancet.

[B30] Green S, Higgins J Cochrane Handbook for Systematic Reviews of Interventions 4.2.5, Section 7.5. http://www.cochrane.dk/cochrane/handbook/handbook.htm.

[B31] Stewart LA, Tierney JF (2002). To IPD or not to IPD?. Eval Health Prof.

