# Supplementary material for: Children in reviews: Methodological issues in child-relevant evidence syntheses
Source: BMC Pediatr. 2005 Sep 21;5:38. doi: 10.1186/1471-2431-5-38 (PMC1261269; doi:10.1186/1471-2431-5-38)
Supplement: Additional File 1 — Appendix A. This is appendix A for the manuscript that provides details of the data extracted from each systematic review included in the study. [file 1471-2431-5-38-S1.doc]

# Appendix A: Items extracted from each review (data were extracted into an electronic database)

# Identifying Information

1. Review Number:
2. First Author:
3. Extractor:
4. Country:
5. Year of Most Recent Amendment:
6. External Funding Agencies:
7. Internal Funding Agencies:
8. Cochrane Editorial Group:

**Review Characteristics**

1. Disease/Condition:
2. Types of included study designs:
3. Types of participants:
4. Age of participants:
5. Intervention(s):
6. Comparison(s):
7. Additional inclusion criteria:
8. What is the primary outcome identified by the review authors?
9. State the first outcome listed in the outcomes section:
10. For the first comparison listed, state the outcome that had the largest number of trials in which it was investigated:
11. Electronic databases searched (list first three listed):
    1. Years searched (for each):
12. Languages of studies searched for:
13. According to the systematic review authors did they search for child studies?
14. According to the systematic review authors did they search for adult studies?
15. Method used to assess quality (list first three listed):

**Results**

1. Language of included studies:
2. Total number of included studies:
3. According to the systematic review authors, were child studies included?
   1. How many?
   2. How did the systematic review authors define a child study (age groups)?
   3. Did they include >18 as children?
4. According to the systematic review authors, were adult studies included?
   1. How many?
   2. How did the systematic review authors define an adult study (age groups)?
   3. Did they include <18 as adults?
5. According to the systematic review authors, were mixed studies included?
   1. How many?
6. According to our definition of children (0-18) how many child studies did they include (refer to table included studies in systematic review)?
7. According to our definition of adults (18+) how many adult studies did they include (refer to table of included studies in systematic review)?
8. According to our definition (0 to >18) how many mixed studies did they include (refer to table of included studies in systematic review)?
9. How many studies are you unable to classify according to our age categories (as a result of them not being listed in the table)?
10. What is the total number of participants included in the review?
    1. Was this reported in the review? Or did the data extractor have to calculate it? Could it be calculated?
11. What is the total number of children (defined by review authors) included in the review:
    1. Was this reported in the review? Or did the data extractor have to calculate it? Could it be calculated?
12. What is the total Number of adults (defined by review authors) included in the review:
    1. Was this reported in the review? Or did the data extractor have to calculate it? Could it be calculated?
13. Were the systematic review authors able to combine for a meta-analysis?
14. Did the systematic review authors consider adverse (complications, etc) events?
15. Did they find any adverse events?

**Subgroup analyses:**

1. Did the authors plan to conduct a subgroup analysis based on age?
2. What rationale, if any, did they provide for conducting an age related subgroup analysis?
3. Did the systematic review authors conduct a subgroup analysis based on age?
   1. What age groups did they use for the adult subgroups?
   2. What age groups did they use for the child subgroup?
4. For mixed reviews where the authors conducted subgroup analyses did the authors note different effects in adults and children?
5. Did they find a statistically significant difference in effect size between adults and children?

**Conclusions**

1. What were the author’s conclusions about the effectiveness of the intervention?
2. What were the author’s conclusions about the effectiveness of the intervention for children?
3. What were the author’s conclusions about the effectiveness of the intervention for adults?
4. Is the condition relevant to both children and adults?
5. Is the intervention relevant to both children and adults?

**Criteria for quantitative analysis for our study**

1. Using the primary outcome, did some of the included studies have enough data to include them in our quantitative analysis (of effect size differences between adults and children)? (This can be determined by looking at the meta-graphs(s))
   1. If so, how many studies?
   2. How many of these were adult only studies (based on our definition)?
   3. How many of these were child only studies (based on our definition)?
   4. How many of these were mixed studies (based on our definition)?
2. Using the first outcome listed, did some of the included studies have enough data to include them in our quantitative analysis (of effect size differences between adults and children)? (This can be determined by looking at the meta-graph(s))
3. If so, how many studies?
4. How many of these were adult only studies (based on our definition)?
5. How many of these were child only studies (based on our definition)?
6. How many of these were mixed studies (based on our definition)?
7. Using the outcomes listed under the first comparison that had the largest number of trials where it was investigated, did some of the included studies have enough data to include them in the quantitative analysis (of effect size differences between adults and children)? (This can be determined by looking at the meta-graph(s))
   - 1. If so, how many studies?
     2. How many of these were adult only studies (based on our definition)?
     3. How many of these were child only studies (based on our definition)?
     4. How many of these were mixed studies (based on our definition)?
